# Co-design and prototype development of the ‘Ayzot App’: A mobile phone based remote monitoring system for palliative care

**DOI:** 10.1177/02692163231162408

**Published:** 2023-03-31

**Authors:** Nicola Carey, Ephrem Abathun, Roma Maguire, Yohans Wodaje, Catherine Royce, Nicola Ayers

**Affiliations:** 1 Department of Nursing and Midwifery, University of the Highlands and Islands, UK; 2 Hospice Ethiopia, Addis Ababa, Ethiopia; 3 Digital Health and Care and Research, University of Strathclyde, UK; 4 Telemed Medical Services, Addis Ababa, Ethiopia; 5 Independent Medical Advisor, UK; 6 Palliative Care Advisor, Ministry of Health, Ethiopia; 7 School of Nursing, BPP University, Waterloo, London, UK

**Keywords:** Ethiopia, palliative care, mHealth, app development, user testing, co-design

## Abstract

**Background:**

Palliative care, a recognised component of care by the World Health Organization is poorly developed in low- and middle-income countries. Mobile phone technology, an effective way to increase access and sustainability of healthcare systems globally, has demonstrated benefits within palliative care service delivery, but is yet to be utilised in Ethiopia.

**Aim:**

To co-design, develop and evaluate a mobile phone based remote monitoring system for use by palliative care patients in Ethiopia

**Design:**

Two-phase co-design approach comprising multiple methods that is stakeholder interviews, focus groups, user-co-creation activities and healthcare worker prioritisation discussions 2019–2020. Phase-1 interviews (*n* = 40), Phase-2 focus groups (*n* = 3) and interviews (*n* = 10).

**Setting/Participants:**

Hospice Ethiopia and Yekatit 12 Medical College Hospital: healthcare workers, palliative care patients, family carers & software-developers.

**Results:**

Co-design activities lead to development of the prototype ‘**Ayzot**’ application, which was well received and reported to be easy to use. Patients, and family caregivers saw provision of self-care information and symptom management as a key function of the App and expressed very positive attitudes towards such information being included. Healthcare workers found the App offered service benefits, in terms of time and cost-savings.

**Conclusion:**

This paper provides a detailed example of the development and design of a prototype remote monitoring system using mobile phone technology for palliative care use in Ethiopia. Further development and real-world testing are required, to not only understand how it acts within usual care to deliver anticipated benefits but also to explore its effectiveness and provide cost estimates for wider implementation.

**What is already known about this topic**

- There is a need to transform palliative care provision in Ethiopia where <1% of 150,000 cancer cases/year receive specialist treatment, due to late presentation and lack of services, and >1.2 million have HIV diagnosis.
- Mobile phone technology (mHealth) in Ethiopia is acceptable and benefits identified but to date no studies have developed a remote monitoring system using mobile phone technology for use by palliative care patients in Ethiopia.

**What this paper adds**

- Development of ‘**Ayzot**’ an Ethiopian based App has been an important first step in the development of an innovative, low cost, sustainable system for palliative care in Ethiopia.
- Users of the prototype App reported high levels of satisfaction, and easily accessible information regarding palliative care symptom management.

**Implications for practice, theory or policy**

- **Ayzot** i) enables palliative care patients and family members to identify problems and initiate necessary treatments earlier, making effective use of limited resources & ii) should alleviate suffering, potentially extending life through optimal symptom control
- **Ayzot** could be the impetus to fundamentally change management of long-term conditions across other Least Developed Countries.

## Introduction

Global level predictions indicate that by 2060 approximately 48 million people will die each year with serious health-related suffering, 83% of which will occur in patients from low and middle income countries.^1,2^ Palliative care, a recognised component of care by the World Health Organization (WHO) improves quality of life and symptom control for those with life-threatening illnesses for example HIV/AIDS and non-communicable diseases.^3^ However, of the estimated 40 million people who need palliative care each year, 78% of whom live in low- and middle-income countries; only 14% who need palliative care currently receive it.^4,5^

Ethiopia, a country of 115 million people has limited health infrastructure.^4,6^ Palliative care provision faces similar issues to the rest of Ethiopia’s healthcare system: poverty, a lack of healthcare professionals, a rural population that makes access difficult and poor transport.^6,7^ Introduced by Hospice Ethiopia a non-governmental organisation, palliative care became part of the government system in 2015^8,9^ with subsequent development of national PC guidelines,^9^ for both hospitals^10^ and primary care in Ethiopia.^11^ Limited services however, mean patients frequently experience palliative care symptoms including pain, breathlessness, and agitation and there is a lack of support for patients and families.^2,3,12^

### Role of eHealth

Information communication technology that is remote or virtual monitoring and consultation services, tools for self-management, and health data analytics, also known as eHealth,^13^ has the potential to transform health care around the world.^14,15^ Benefits of eHealth include enhanced delivery of cancer care through improved patient-provider communication, symptom control, and optimised patient engagement across the cancer care continuum.^16,17^ A significant reduction in symptom burden and symptom experience was recently reported in a large European randomised controlled trial (eSMART).^17^ Likewise palliative care has also seen increasingly deployment and benefits of eHealth for patients in the United Kingdom and African countries with positive effects on information sharing, education, decision making, communication and costs.^13,18–20^

### Role of mHealth in Palliative Care

mHealth, the use of mobile devices including phones, provides a significant opportunity to support patients and improve access to palliative care^14^ particularly in large rural regions of Africa where until now only limited access was available.^18,21–24^ Recognising the implications and potential of using technology to improve access to healthcare in Ethiopia a national eHealth strategy was recently developed.^19,24^ Evidence suggests that mHealth in Ethiopia has a positive effect on HIV/AIDS and maternal services,^18^ and more recently women with cervical cancer.^19^ Existing mHealth projects in Ethiopia have highlighted challenges around a lack of eHealth literacy in health extension workers; and a lack of cultural and language considerations where mobile phone applications (Apps) have been developed in English only.^25^ Barriers such as cost, coverage, and knowledge of using the technology also remain problematic.^22,23^ The importance of understanding preferences and priorities of palliative care patients, family carers and healthcare workers through robust user engagement has also been highlighted.^26,27^

Preliminary work undertaken in Ethiopia by the authors (2015–2017) identified a high level of interest in using technology to support palliative care by healthcare.^28^ Research to date suggests mobile phone technology in Ethiopia is acceptable^18,19^ and the need to develop an mHealth platform to improve palliative care services recognised.^24^ However, to date no studies have developed a remote monitoring system using mobile phone technology for use by palliative care patients in Ethiopia.

## Aim

To co-design, and develop a mobile phone based remote monitoring system for use by palliative care patients in Ethiopia with key stakeholders (palliative care patients, healthcare workers, family carers & software-developers).

### Objectives

*Phase 1*: to identify patients and stakeholder perspectives on the use of mobile phone technology to support palliative care that is information and support needs, and specific symptoms that should be assessed and design/presentation user interface.

*Phase 2*: to explore patients and stakeholder perceptions and user experience of the App prototype

## Methods

### Study design

**Figure 1.**
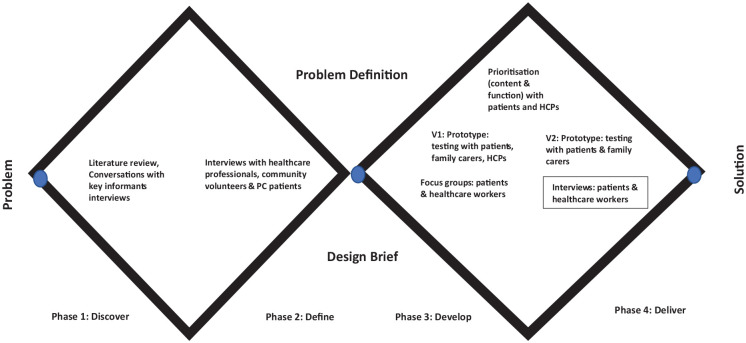
Study methods with double diamond model.

Informed by the Medical Research Council’s Framework for development, evaluation and implementation of complex interventions,^29^ and World Health Organization^30^ recommendations a co-design approach was adopted to ensure active involvement of stakeholders as advocated for the design and evaluation of new technologies in health care.^31–33^ The Design Council’s Double Diamond model was also utilised to support a participatory co-design approach, and creation of several possible ideas (‘divergent thinking’) before refining and narrowing down to the best idea (‘convergent thinking’) (see Figure 1).^34^

**Figure 2.**
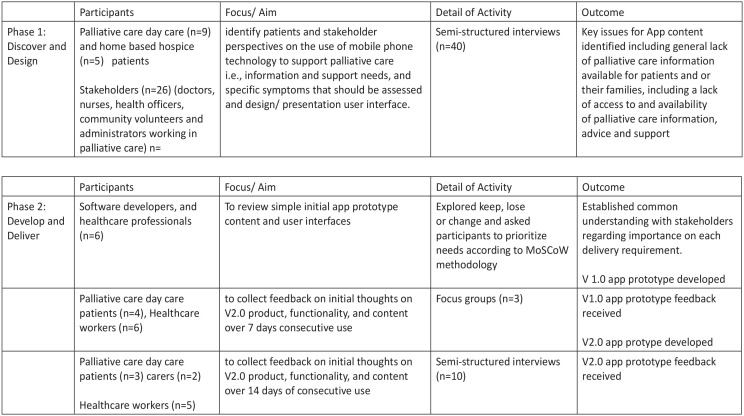
Overview of the co-design process.

Focussing on end users that is palliative care patients and the need for a usable, meaningful and accessible mobile phone application, multiple user-centred methods were used throughout a two-stage iterative process (see Figure 2):

## Ethics and consent

Ethical approval was granted from: i) University of Surrey Ethics Committee (UEC2019 042 FHMS) and ii) Hospice Ethiopia and Yekatit 12 Medical College Hospital, Ethiopia. Informed written consent was obtained prior to participation; May 2019–August 2020.

### Study methods

#### Phase 1: Discover and define: Interviews with patients and stakeholders

*Sample*: A purposive sample of doctors, nurses, health officers, community volunteers and administrators working in palliative care and day care palliative care patients from Hospice Ethiopia (main palliative care NGO in Ethiopia) and Yekatit 12 Medical College Hospital (a government hospital with palliative care hub), Addis Ababa.

*Recruitment*: We contacted known healthcare workers who had an interest in improving palliative care provision. Day care patients who expressed an interest in sharing their views and experiences of palliative care were approached by locally based researchers to participate. A standard process of informed consent was conducted prior to undertaking semi-structured interviews.

*Data collection*: Interviews explored four main areas related to palliative care^1,8,19^: i) information, advice and support received; ii) challenges accessing information, advice, and support for patients; iii) challenges for family members/friends supporting; iv) use of mobile phone technology and potential use for palliative care in Ethiopia.

*Data analysis*: Interviews (*n* = 40) (see Table 1) were subsequently transcribed and translated by an experienced bi-lingual Amharic-English translator (FA). Two interviews were back translated (FA) and found to be accurate (NA, FA) prior to thematic analysis. Following familiarisation, data were analysed by generating descriptive, process, structural and in-vivo codes, categories and finally themes (NA). The development of codes and themes were discussed with EA and the other data collectors to check for accuracy. Coding was done using descriptive, process, structural and in vivo coding.^35^

**Table 1.** Phase 1: participant information.

*N* = 40	Number of participants
Participant group	
Patients	14
Nurses	8
Doctors	5
Community volunteers	5
Health officers	5
Administrators	3
Setting	
Non-governmental organisation (NGO)	28
Hospital	12

#### Phase 2: Develop: Development of App prototype version 1.0 and 2.0 (co-design activitie; focus groups and interviews with patients and hospice staff)

*Sample*: A co-design group: comprising 36 participants at Hospice Ethiopia who were willing to contribute to the co-design group and discussions was established. Members of the co-design group were invited to engage in a series of discussions facilitated by locally based researchers to prioritise app content and functionality, explore usability, general views and acceptability of App prototype version 1.0 and 2.0.

*Recruitment*: Local researchers approached day care patients during routine visits at the hospice (*n* = 26) and hospice staff (doctors, nurses, community volunteers and administrators) (*n* = 10): those who expressed an interest in contributing to the study were invited to participate. Inclusion criteria were that participants were aged over 16, and able to commit up to 4 hours in total to the co-design process.

##### App development and testing

Incorporating information gathered in the ‘Discover and Define’ (phase 1), a prototype of the newly named ‘**Ayzot**’ App including content and presentation on user interfaces was developed. ‘**Ayzot**’ means reassurance in Amharic and the name identified during informal conversations between the software development team (*n* = 3) and members of the co-design group. The prioritization exercise was facilitated by the four categories of the ‘MoSCoW’ co-design approach (must have, should have, could have and won’t have),^36^ a recognised technique to achieve common understanding with stakeholders regarding importance on each delivery requirement.

Members of the co-design group (day care patients (*n* = 10) and hospice staff (*n* = 6)) were invited to use the app for seven consecutive days and participate in subsequent focus groups (*n* = 3) to discuss relevance of content, ability to understand the questions, current and future app content; suggestions for improvement, and App features they would like to keep, change or loose (Table 3).

A medium-fidelity closed system prototype V2.0 of the **Ayzot** application was subsequently developed (See Supplemental file 1 for App overview and content) providing limited functionality, and one way communication, but with clickable areas which presents the interactions and navigation possibilities of the proposed live application. Time constraints meant it was not possible to test using a live system and hence it was not as interactive an originally intended: however, it addressed most key features and functionality identified in previous phases as to enable closed-system user testing.

A further cohort of patients (*n* = 10) and hospice staff (*n* = 5) from the established co-design group at Hospice Ethiopia were invited to use the second iteration of the app prototype for 14 days (six of whom had used version 1.0). A final set of interviews (*n* = 10) (Table 4) was conducted to collect feedback from participants regarding initial thoughts on functionality, content, and product acceptability. Although previous participation in the study may have affected their views, their more in-depth understanding could also have made greater insight into the experience.

*Data analysis*: Interviews were audio-recorded to aid framework analysis,^37,38^ and followed phase 1 procedures for transcribing, translation and back translation to ensure content accuracy (NA, FA). A long version of the framework which incorporated all the participant feedback on the experience of using the app, appearance and usability, availability of appropriate palliative care information and if further advice or information was required was created. This was then shortened and developed into an analytical framework to facilitate description of the current functionality and acceptability of the application in the home setting thus allowing logical conclusions to be drawn to support future changes.

*Ethics*: As per standard ethical procedures, informed consent was obtained prior to participation in each stage of the co-design process

## Results

*Phase 1*: Fourteen patients, and 26 stakeholders across two sites participated in interviews (see Table 1). Most patients (*n* = 9, 64%) accessed care as day/outpatients, and five (36%) the home-based hospice service.

Patient and stakeholders identified similar issues regarding limitations of current palliative care provision and symptoms. *Key symptoms* such as pain, nausea, fatigue, drowsiness and advice re self-management especially vomiting, diarrhoea, breathlessness and wound care as well as issues regarding dietary intake were highlighted. Having access to information for family members to support patients, where to get help and education regarding medicines were also acknowledged as important by patients, community volunteers and healthcare professionals. Additional concerns such as emotional well-being and spiritual care needs were identified as being important. There was consensus regarding general lack of palliative care information available for patients and or their families, including a lack of access to and availability of palliative care information, advice and support. Despite limited use of apps to date, particularly by patients, there was agreement regarding numerous potential benefits participants felt a mobile-phone application could provide.

Key issues for inclusion were identified and used to develop and inform the next step which was to develop App prototype content for use in part 2 (see Supplemental file 1 for details). Key issues (see Table 2) demonstrate some were identified by both patients and stakeholders, whereas others were only identified by one group. Healthcare workers tended to identify issues of a more physical nature related to pain management and likely to cause distress to patients, whereas those identified by patients tended to focus on psychological aspects of care and accessing information.

**Table 2.** Symptoms/issues identified as important for V1.0 App prototype.

Patients and Healthcare workers	Patient only	Healthcare workers
Symptom assessment and management that is pain, nausea, vomiting, diarrhoea How to take medicines Self-management information Spiritual information & contact details Help patients who ‘hiding’ do not access palliative care	Dietary advice Information and support contact details Information for family members	Medicines information Wound care information Morphine diary use Record vital signs Use of videos to support content Multiple disease information Not to be used to ask about patient feelings

### Phase 2

i) *App prototype V1.0*: focus groups with patients (*n* = 4) and staff (*n* = 6) explored patients and healthcare workers perspectives about the various sections of App V1.0 and thoughts on how it could be developed and improved. There were no major differences in opinions and perspectives relative to the prototype (see Table 3).

**Table 3.** Patient and healthcare workers perspectives on App prototype V1.0.

App content	Patients	Healthcare workers
Physical symptoms	All questions relevant and understandable, prefer use of the word chronic rather than incurable illness Presentation: no specific preference Ordering: no specific preference re symptoms Adding questions regarding symptoms of fever, cough, sweating, vomiting, and abdominal cramp or option for ‘any other symptoms’ Add advice and information for other conditions such as diabetes and hypertension	Preference to focus on main symptoms for example pain, nausea, breathlessness, vomiting, diarrhoea For other symptoms use App to promote self-care and providing background information and advice
Emotional issues	Spiritual advice is good as covers three major religions Frequency; would vary depending on symptoms and prognosis; might be helpful for seeing change over time	Frequency – helpful to consultations to understand how patient has been feeling and current concerns
Functionality	Self-care management: Very helpful for example ‘it feels like the doctor is beside me’; ‘the app helped me self-manage my symptom until I meet hospice team’, ‘you have the information with you all the time’ Feedback chart: Symptom chart/diary add a description to this to help understanding Other helpful information: change the picture describing pain; add picture for wound care. Include as much information as possible on cancer/HIV/AIDs as not easily available Is it possible to have audio recordings for those who can’t read? Language: minor language/translation formatting issues as App is available in English and Amharic	Self-care information Good to provide information to support self/care self-management for patient and family members Amharic language is too formal – need to be colloquial Feedback reports/graphs; daily/weekly summaries would be helpful for both patients and clinicians
Alerting functions	Chart: rename *symptom information for your healthcare worker* or something to this effect. Add text to this section advising that-this information can be used to show your healthcare worker how you have been doing or something to this effect	Helpful to have a chart showing symptoms and experience over time Remote alerts and link up to healthcare professionals in the future would beneficial
Issues to consider	Could the app be personalised to record whether it is the patient or carer who completes the content Some introductory text and direction would be helpful The app is reassuring when I do not have access to the healthcare worker	Need to use simple language and pictures where possible Benefits more than just the patient

ii) *App prototype V2.0*: Interviews (*n* = 10) (patients *n* = 3, carers *n* = 2, and healthcare workers *n* = 5) (Table 4) were conducted to collect feedback on initial thoughts on V2.0 product, functionality, and content. Participants were aged 30–50 years. Patients had a diagnosis of cancer or HIV/AIDS, and healthcare workers 3–15 years palliative care experience. Covid-19 visiting restrictions meant it was not possible to collect data from the remaining five patients who used the App.

**Table 4.** Part 2 interview participant information.

*N* = 10	Number of participants
Patients	3
Family carers	2
Doctors	2
Community volunteers	1
Administrator	1
Nurse	1

User perceptions in terms of interface, acceptability, areas of development and thoughts on the advantages of such an application are presented in Table 5. These perceptions show that the App V2.0 was well accepted, easy to use, well created and offered many advantages for palliative care patients, family carers and healthcare workers. Patients continued to highlight the importance of cultural considerations and need for spiritual care information. Healthcare workers prioritisation exercise identified the top four priorities for App content were self-care, physical symptom assessment, emotional well-being and information giving, whereas top four priorities for the app function were symptom monitoring, identifying, and recording change in symptoms, promotion of self-care and improving patient experience.

**Table 5.** Perspectives on App prototype V2.

User interface design	Attractive graphics, use more pictures and less text; would be good to able edit diary notes; can photos be of Ethiopians rather than people with same colour skin Font size – can this be changed/edited so can be changed by user. Some minor language issues that need to be addressed Previous feedback has been incorporated into this version
Acceptance	Very simple and easy to use for educated and non-educated people Content very relevant and used by whole family, can be used at anytime Lot of benefits – in terms of improving knowledge, provides readily accessible information that supports decision making Good information on spiritual care, wound care, medicines and other symptoms such as pain, nausea Used multiple time each day during testing period
Future consideration	Content: Provide information on constipation, side-effects, and drug interactions from medicines, exercise, more spiritual advice, cultural medicine Having a care-giver version Add in more signposting to help patient/carer use the App, navigating back and forwards around different sections, frequency of use More pictures and less text
Advantages	Very helpful – provides information that I did not realise would be helpful to know It contains everything I need to know in a friendly way Good information, makes patients feel knowledgeable, community now asking me for information Comfortable for patients and carers to use Have learnt a lot and been able to share that knowledge with my family and community Content is helpful and does work in terms of symptom control Addresses palliative care holistically Helpful self-assessment and diary to monitor symptoms Increases confidence for family members During Covid-19 has been very helpful due to restricted visits Helpful if not able to access hospital or healthcare professionals, for those with smart phones in the city and countryside Helps clinicians with time management-cost benefits

## Discussion

### Main findings

As far as we are aware, this is the first study to develop a prototype app to support palliative care needs of patients in Ethiopia. Adopting a co-design approach, the app was informed throughout by patients and healthcare workers. Facilitated by the Design Council’s Double Diamond Model,^34^ a detailed account of each stage of development is provided. This is important as i) it provides an example of a successful method through which to develop a digital health intervention in a Least Developed Country and ii) previous studies have highlighted how app innovations have not been well received when user feedback has not been considered from the start.^25^

There was agreement among patients and healthcare workers in terms of the key symptoms and issues in palliative care provision including pain, nausea, fatigue, drowsiness, and access to relevant information. These results are in line with commonly reported symptoms and issues for palliative care patients.^2,12^ They also reflect the emerging nature of palliative care in Ethiopia and other least developed countries, limited services and lack of information for patients, families and or healthcare professionals.^2,4,7^ It is however, important to note the differences between patients and healthcare workers in relation to some of the symptoms and issues that each group felt should be included with healthcare workers focussing on physical symptoms whereas patients identified spiritual care needs as a priority. This reflects recent concerns regarding Ethiopian nurses and healthcare workers poor levels of palliative care knowledge and understanding.^3,12^ It is possible that using as an App such as this would therefore also improve healthcare workers knowledge in other least developed countries regarding the importance of addressing the holistic care needs of palliative care patients that can affect their overall quality of life and symptom control.^3,8,13,27,39^

Patients and health care workers saw provision of self-care information and symptom management as a key App function, and patients and family caregivers expressed very positive attitudes towards such information being included. This is in line with goals 9,10 and 17 of the 2030 sustainable development agenda which identify the need to reduce inequality within countries^37^ and WHO eHealth strategy.^24^ Although recent advances in palliative care, such as palliative care Hub and spoke services and palliative care guidelines have been established,^9–11^ our findings show that there is still a lack of information for patients and their families. Important cultural issues were revealed,^40,41^ such as patients potentially not being aware of their diagnosis and the ‘hidden patient’. As need continues to outstrip capacity for palliative care services, our study emphasises the need for urgent and rapid implementation of innovative and novel approaches, such as mHealth to help strengthen Ethiopian service^2,4,26^ and other least developed countries.

### What this study adds

Participants were generally very positive about the usefulness of the App, seeing it as a tool that could support both them and their family members. This is in line with recent evidence that indicates E-user satisfaction, in terms of meeting and exceeding expectations, is key to understanding continued app usage intention and behaviour.^42,43^ Interestingly, despite a lack of e-health literacy and previous app usage,^43^ our results suggest that participants motivation to access knowledge and improve their symptoms was strong enough to overcome challenges posed by initial App usage. Limited availability of palliative care information and services in Ethiopia may therefore have a positive effect on technology acceptability and future ‘Ayzot App’ usage. Such positive attitudes should however, not be assumed to translate into sustained use as technology acceptance is complex and often non-linear.^44^ Users’ journey with technology can for example, be affected by different stages, and the extent to they have previously integrated technology into their lives, with many who initially decide to try an application not necessarily using in the long run.^45^

In line with previous review findings,^46^ healthcare workers found the App offered service benefits, in terms of time and cost-savings. Cost savings were felt to arise due to improved responsiveness through increased communication between patients, families and healthcare workers, improved symptom management and ability to prioritise cases requiring a home visit. Symptom control quality of life, and supportive care needs are important components of palliative care provision,^47^ it is however important to note that the App prototype did not include any objective measures. Furthermore, patients and healthcare workers in our study did not identify any desire regarding inclusion of real-time location systems and patient tracking used in Apps to support patients with cancer in other parts of the world.^17^ The ongoing Covid-19 pandemic, at its early stages during app development and data collection in 2020, highlighted the importance of having access to well designed, usable digital systems to remotely support palliative care patients.^48^ While it is anticipated that all potential users, patients, family carers and healthcare workers alike will be more motivated than ever to engage with such opportunities going forwards, partnerships with local governments and non-governmental organizations to secure funding, leadership, and necessary infrastructure need to be established to increase the chance of long-term success.^46^

### Strengths and limitations

A key strength was the user centred methodology that was adopted. This supported a bottom-up approach to developing App content and functionality. Focusing on end-users, our co-design approach ensured patients and healthcare workers were included at each stage of the process.^31–33^

Covid-19 restrictions during data collection in Part 2 meant we were only able to gather feedback on prototype V1 and V2 from Hospice Ethiopia staff and patients, as such there is a lack of understanding regarding App content and functionality by other groups of palliative care patients and healthcare workers in Ethiopia.^30,49^

Some features, for example software log of activity, were not included as project time constraints meant App development between V1 and V2 was limited compared to original study intentions. We were therefore not able to assess user uptake and interaction.^30^ Delays to initial software development meant the prioritisation exercise was only conducted with healthcare workers, not patients, so it was not possible to make a comparison between these two groups.^36^ A more structured method of evaluation in terms of assessing usability would have added reliability to patient evaluation.^29^ The next phase would therefore: i) support real world testing of the **Ayzot** App to assess benefits, risk and unintended consequences; ii) fully develop the app, in line with MRC complex intervention framework, in relation to key components identified by patients and healthcare workers and explore feasibility and acceptability to all users.^29^

## Conclusion

This paper provides a detailed example of the development and design of a remote monitoring system using mobile phone technology for palliative care in Ethiopia. It is anticipated the **Ayzot** app should enable palliative care patients and family members to identity problems sooner and initiate treatments earlier, alleviating suffering and potentially extending life through optimal symptom control. Further development and real-world testing are required, not only to understand how it acts within usual care to deliver anticipated benefits but also to explore effectiveness and cost estimates for wider implementation.

## Supplemental Material

sj-docx-2-pmj-10.1177_02692163231162408 – Supplemental material for Co-design and prototype development of the ‘Ayzot App’: A mobile phone based remote monitoring system for palliative care Click here for additional data file. Supplemental material, sj-docx-2-pmj-10.1177_02692163231162408 for Co-design and prototype development of the ‘Ayzot App’: A mobile phone based remote monitoring system for palliative care by Nicola Carey, Ephrem Abathun, Roma Maguire, Yohans Wodaje, Catherine Royce and Nicola Ayers in Palliative Medicine

sj-pdf-1-pmj-10.1177_02692163231162408 – Supplemental material for Co-design and prototype development of the ‘Ayzot App’: A mobile phone based remote monitoring system for palliative care Click here for additional data file. Supplemental material, sj-pdf-1-pmj-10.1177_02692163231162408 for Co-design and prototype development of the ‘Ayzot App’: A mobile phone based remote monitoring system for palliative care by Nicola Carey, Ephrem Abathun, Roma Maguire, Yohans Wodaje, Catherine Royce and Nicola Ayers in Palliative Medicine
