# Supplementary material for: Co-design and prototype development of the ‘Ayzot App’: A mobile phone based remote monitoring system for palliative care
Source: Palliat Med. 2023 Mar 31;37(5):771–81. doi: 10.1177/02692163231162408 (PMC10227095; doi:10.1177/02692163231162408)
Supplement: sj-docx-2-pmj-10.1177_02692163231162408 – Supplemental material for Co-design and prototype development of the ‘Ayzot App’: A mobile phone based remote monitoring system for palliative care [file sj-docx-2-pmj-10.1177_02692163231162408.docx]

# **Appendix S1 – Reflexivity Statement**

1. **How does this study address local research and policy priorities?**

This study addresses the need to transform palliative care in Ethiopia, an emerging area of policy and service provision. Research and policy are pillars of the Public Health Model that Ethiopia has adopted for developing and improving PC services within the country.

1. **How were local researchers involved in study design?**

The first category of local researchers involved were those with extensive experience of developing and delivering PC services within Ethiopia (NA, EA), specifically NA has experience of working with the Ethiopian Ministry of Health, and EA , Executive Director of Hospice Ethiopia, has previously organised international collaborations.

The second category of local researchers were qualified healthcare professionals who supported study proceedings leading on data collection and assisting with data analysis, interpretation and dissemination (EA, HM, HA) and software development (YW).

In addition there were high income country researchers with experience of conducting, leading or organising research collaborations involving low and middle income countries (RM, CR, NC). There were high income countries researchers with ongoing experience as members of editorial teams (RM, NC). One of the authors, NA although originally from a high-income country has worked with diverse cultures in low-income countries for over 25 years, making significant contributions to the organisation and leadership of multiple international collaborations.

1. **How has funding been used to support the local research team?**

This project was used to support the local research team by donating funds to Hospice Ethiopia (a local Non-Governmental Organisation) to facilitate study processes, along with supporting staff and patients, and the Ayzot App by an Ethiopian based software development company (YW). The funding was also used to support local delivery of research skills workshops to EA, HA, HM. This led to EA wanting to pursue his research career and a successful application to undertake a PhD.

1. **How are research staff who conducted data collection acknowledged?**

All members of the project team are included as authors. Each team member was designated and delivered specific role(s) during the writing process (see acknowledgements). Local researchers who contributed to data collection, and translation are also acknowledged in the article.

1. **Do all members of the research partnership have access to study data?**

All members of the partnership have access to data.

1. **How was data used to develop analytical skills within the partnership?**

Team members worked together in small groups and as part of the project advisory group to leverage multidisciplinary skills with an aim to develop language and content accessible to a broad range of end users.

1. **How have research partners collaborated in interpreting study data?**

Two inclusive workshops were held during the process of developing the application. These workshops included research methods training but also involved considering the data, specific cultural issues along with linguistic differences and interpretation. Furthermore, the translator (FA) also was involved in interpreting cultural aspects of the data. Interim findings were presented to the project advisory group and Ethiopian based patient and public involvement group where participants collaborated to agree on recommendations and content of the reflexivity statements.

1. **How were research partners supported to develop writing skills?**

The research team writing this statement is predominantly composed of senior academics. Local researchers on the authorship team (EA, YW) were supported to develop and refine their writing skills within the working groups.

1. **How will research products be shared to address local needs?**

Study findings were presented at international conferences 2019-2021 and at the African Palliative Care Conference in August 2022.

Dissemination events include publication of the article as open access, and cascading details via Ethiopian based palliative care website [https://ethiopiapalliativecare.wordpress.com](https://ethiopiapalliativecare.wordpress.com/) along with social media including Twitter, LinkedIn, and University webpages.

Additional activities include engagement with research leaders in global health and other fields involved in international collaborations, and with journalists based in high-income countries and low- and middle-income countries.

1. **How is the leadership, contribution and ownership of this work by LMIC researchers recognised within the authorship?**

The authorship team comprises all project team members, three of whom live in a low-income country and three high-income. Author NA, although originating from a high-income country, has lived and worked in low-income countries for over 25 years. Each team member was designated and delivered specific role(s) during the writing process (see acknowledgements). NA was part of the senior authorship team in developing this manuscript, and this contribution has been recognised as being named last author. The primary reason for this is to provide an opportunity for each team member to make an active contribution to the writing and development of the manuscript.

1. **How have early career researchers across the partnership been included within the authorship team?**

The authorship team comprises all project team members, only one of whom is an early career researcher. They attended the research workshops, and contributed to data collection, analysis, interpretation and development of the App.

1. **How has gender balance been addressed within the authorship?**

Four authors are female (NC, NEA, RM, CR,) and two authors are male (EA. YW)

1. **How has the project contributed to training of LMIC researchers?**

Two inclusive workshops were held during the process of developing the application. These workshops included research methods training but also involved considering the data, specific cultural issues along with linguistic differences and interpretation. Interim findings were presented to the project advisory group and Ethiopian based patient and public involvement group where participants collaborated to agree on recommendations and content of the reflexivity statements.

1. **How has the project contributed to improvements in local infrastructure?**

This project has not directly contributed to improvements in local infrastructure, but it is envisaged after further testing it will support the Ministry of Health and other service providers to improve palliative care service provision.

1. **What safeguarding procedures were used to protect local study participants and researchers?**

Ethical approval was granted from the University of Surrey Ethics Committee (UEC2019 042 FHMS). Permission was further sort and granted in Ethiopia from Hospice Ethiopia and Yekatit 12 Medical College Hospital. Informed consent was obtained prior to participation between May 2019- August 2020.
