# Supplementary material for: Co-design and prototype development of the ‘Ayzot App’: A mobile phone based remote monitoring system for palliative care
Source: Palliat Med. 2023 Mar 31;37(5):771–81. doi: 10.1177/02692163231162408 (PMC10227095; doi:10.1177/02692163231162408)
Supplement: sj-pdf-1-pmj-10.1177_02692163231162408 – Supplemental material for Co-design and prototype development of the ‘Ayzot App’: A mobile phone based remote monitoring system for palliative care [file sj-pdf-1-pmj-10.1177_02692163231162408.pdf]

Supplementary File 1: App overview and content

App introduction and menu options	User options	Language options

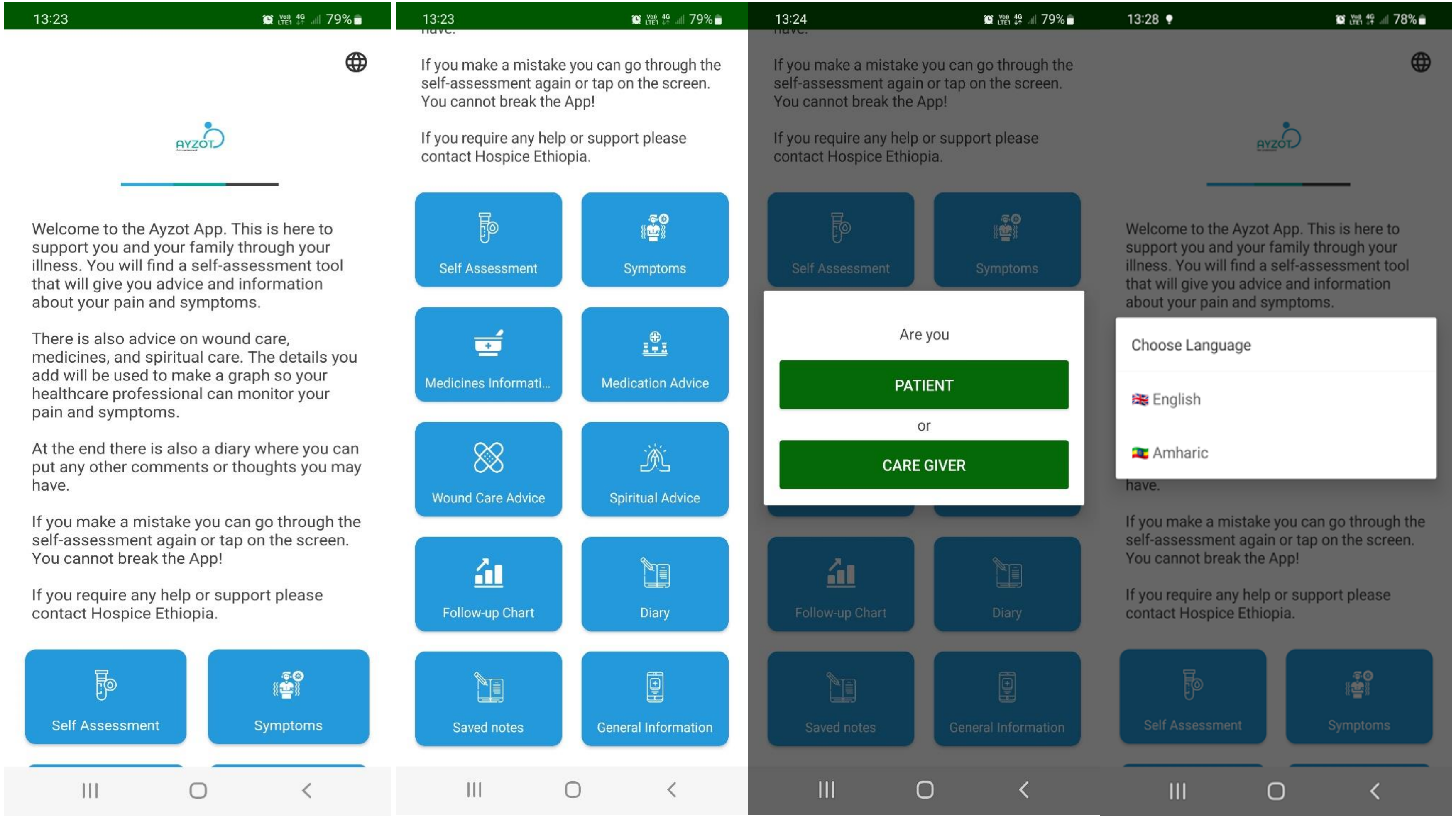

Supplementary File 1: App overview and content

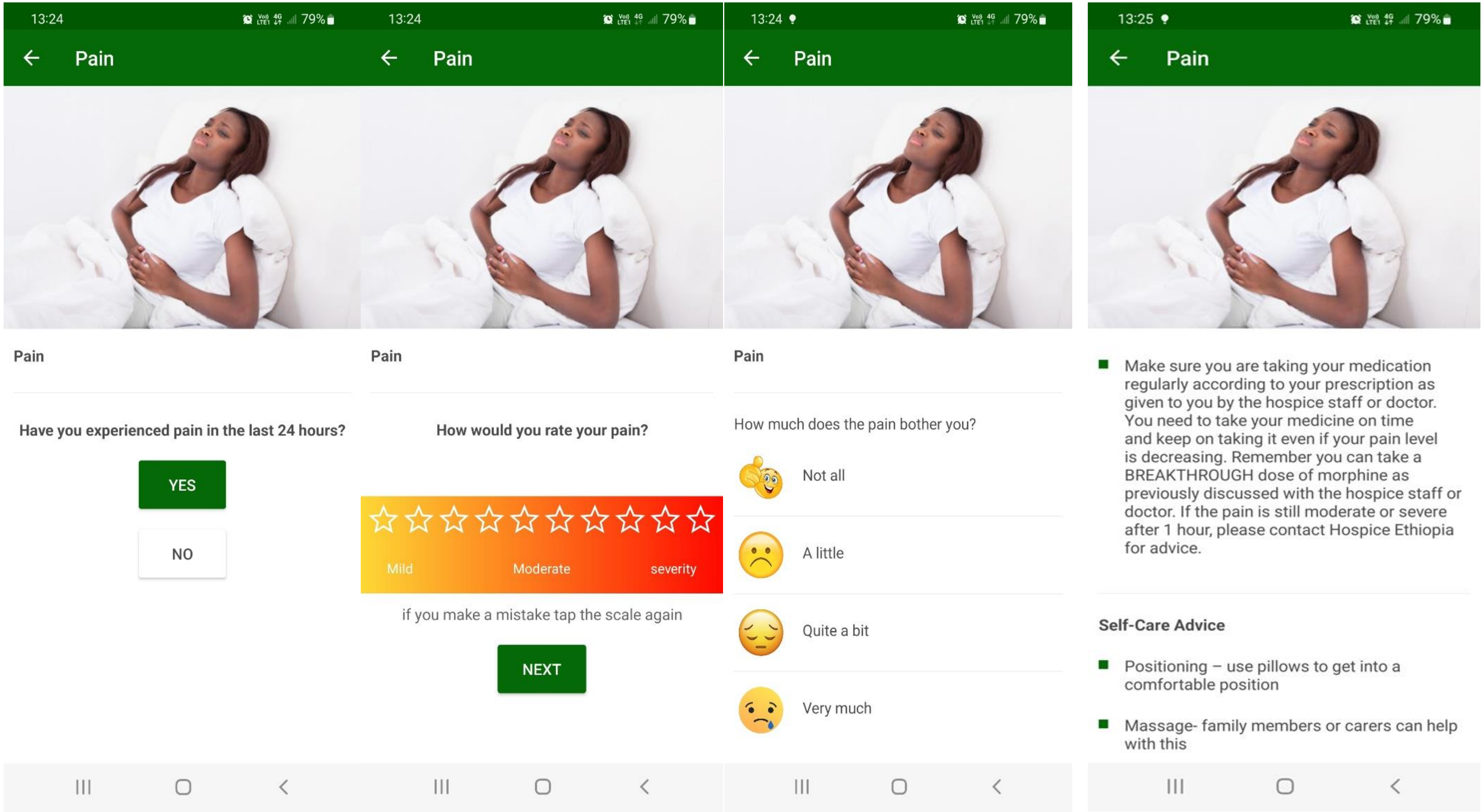

**Example of symptom assessment**
Pain

Supplementary File 1: App overview and content

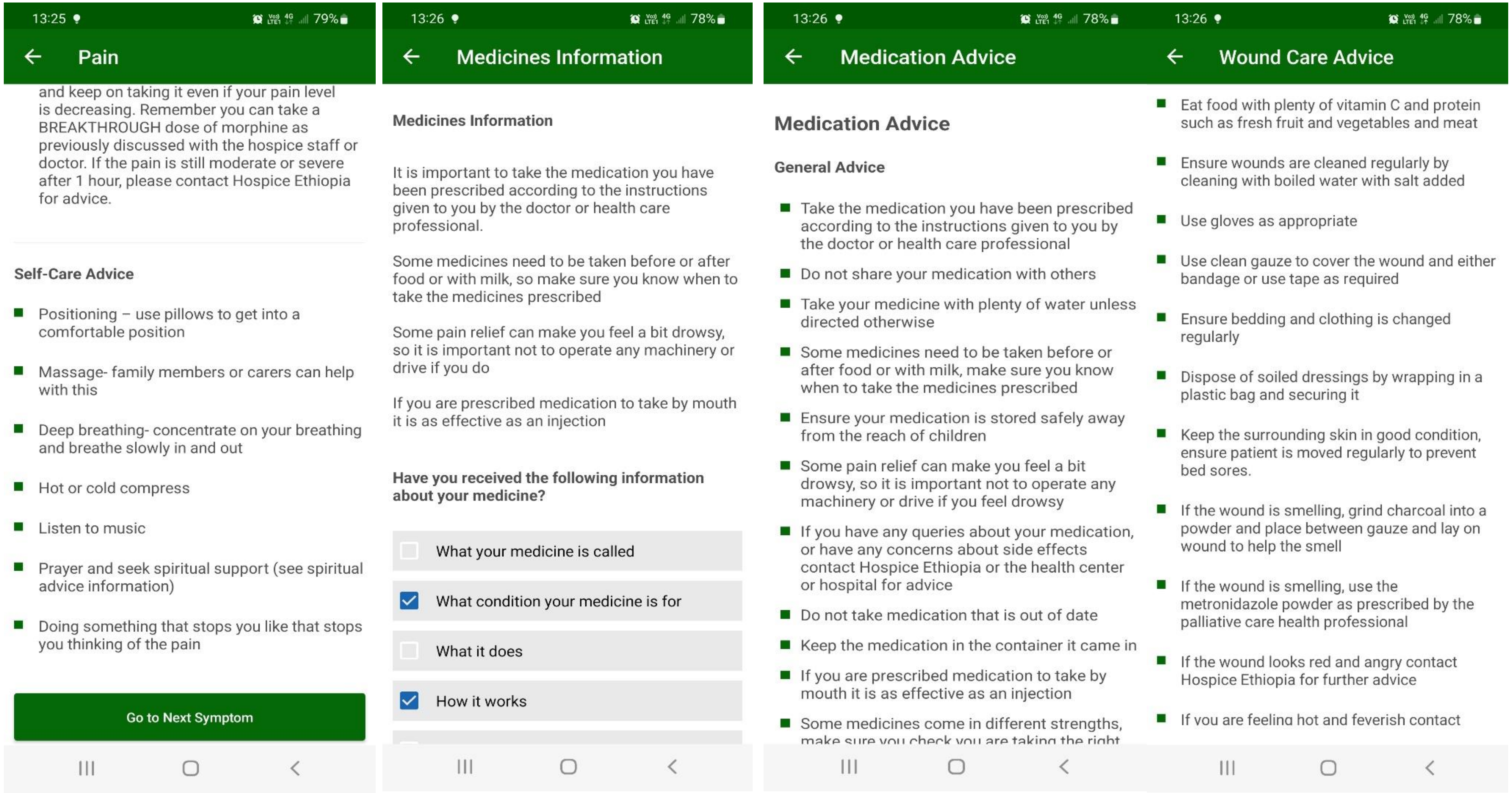

Examples of advice and information			
1. Self-care advice: Pain	2. Medicines information	3. Medication advice	4. Wound care advice

Supplementary File 1: App overview and content

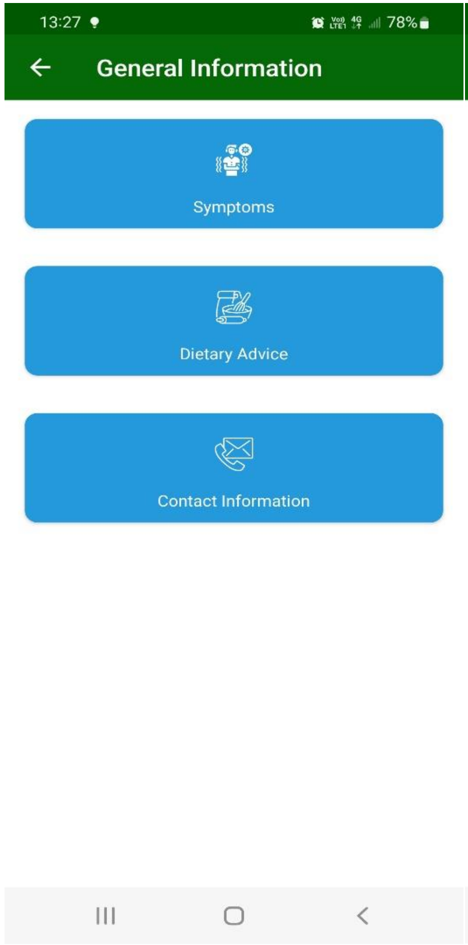

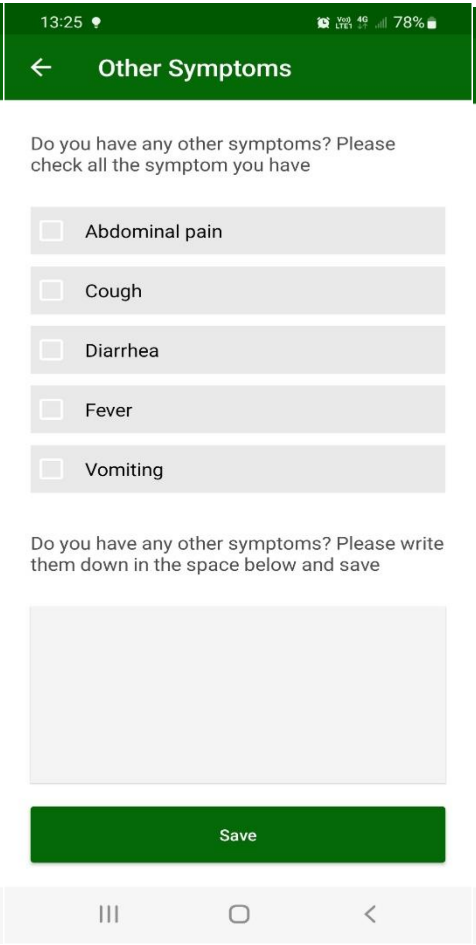

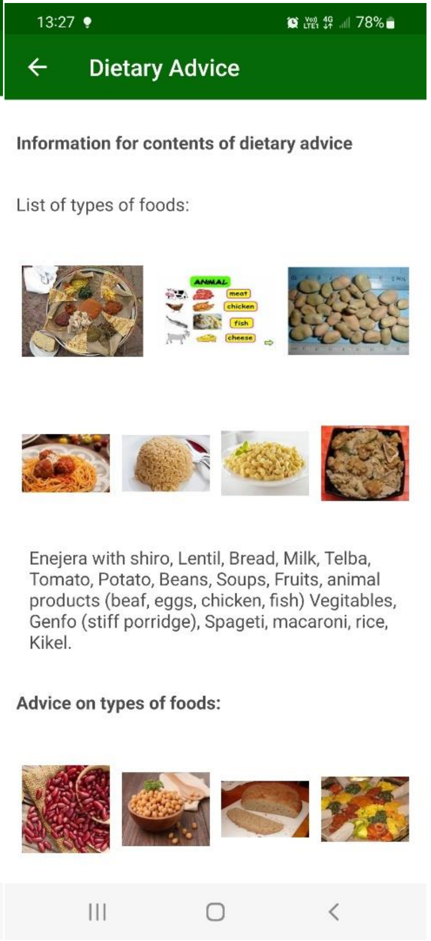

**General information menu options**	**Assessment of other symptoms**	**Dietary advice**

Supplementary File 1: App overview and content

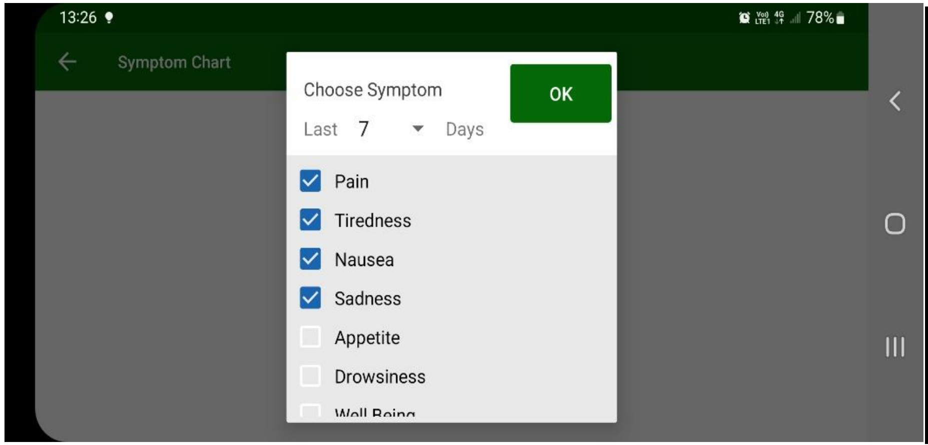

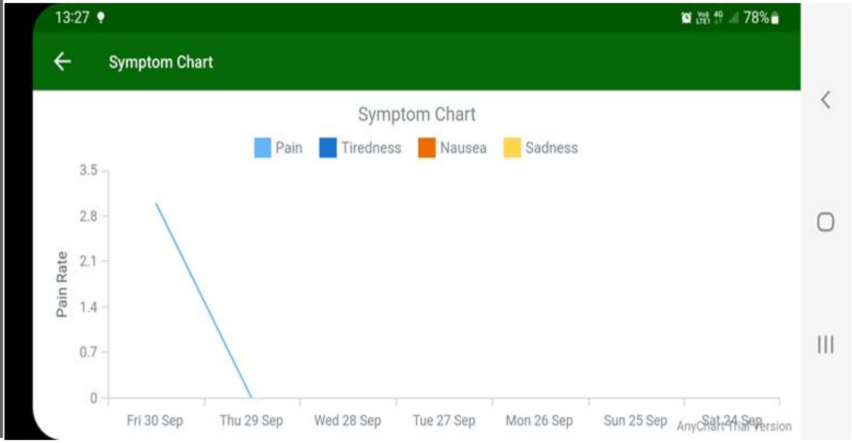

Symptom chart- reflecting record of symptom assessment over last 7-30 days	
Symptom menu options	Symptom chart
